# Cardamonin Regulates miR-21 Expression and Suppresses Angiogenesis Induced by Vascular Endothelial Growth Factor

**DOI:** 10.1155/2015/501581

**Published:** 2015-07-21

**Authors:** Fu-Sheng Jiang, Sha-Sha Tian, Jin-Jian Lu, Xing-Hong Ding, Chao-Dong Qian, Bin Ding, Zhi-Shan Ding, Bo Jin

**Affiliations:** ^1^College of Life Sciences, Zhejiang Chinese Medical University, Hangzhou, Zhejiang 310053, China; ^2^State Key Laboratory of Quality Research in Chinese Medicine, Institute of Chinese Medical Sciences, University of Macau, Macau

## Abstract

Cardamonin has promising potential in cancer prevention and therapy by interacting with proteins and modifying the expressions and activities, including factors of cell survival, proliferation, and angiogenesis. In our precious study, we have demonstrated that cardamonin suppressed vascular endothelial growth factor- (VEGF-) induced angiogenesis as evaluated in the mouse aortic ring assay. It is also known that microRNAs (miRNAs) play important roles in angiogenesis. Herein, we hypothesized whether antiangiogenesis effect of cardamonin in human umbilical vein endothelial cells (HUVECs) triggered by VEGF was associated with miRNAs. We found that cardamonin reduced the miR-21 expression induced by VEGF in HUVECs. Treatment with miR-21 mimics abolished the effects of cardamonin on VEGF-induced cell proliferation, migration, and angiogenesis in HUVECs. However, treatment with miR-21 inhibitors presented the opposite effects, indicating the vital role of miR-21 in this process. Our study provides a new insight of the preliminary mechanism of anti-VEGF-induced angiogenesis by cardamonin in HUVECs.

## 1. Introduction

Cardamonin is a chalcone that belongs to the flavonoid family isolated from many kinds of herbs, such as* Alpinia katsumadai*,* Carya cathayensis *Sarg. [1],* G. biloba* [2], and* Gynostemma pentaphyllum* [3], and is often responsible for the yellow pigmentation of plants [4, 5]. As shown by the increasing number of publications, cardamonin has attractive scientists' attention due to its benefits to human health. It presents various pharmacological activities, including anti-inflammatory [6, 7], antineoplastic [8], antioxidant [9], and anti-infectious [10] properties. Cardamonin inhibits smooth muscle cell proliferation and migration [11, 12], prevents endothelial barrier dysfunction [13], and suppresses vascular endothelial growth factor- (VEGF-) induced angiogenesis as evidenced by the mouse aortic ring assay [14].

Excessive angiogenesis (neovascularization) is the characteristic of a number of serious diseases, including cancer [15], rheumatoid arthritis [16], and atherosclerosis [17]. In our previous study, we found the total flavonoids (TFs), isolated from the leaves of* Carya cathayensis *Sarg., and five flavonoids of the main components of the TFs, that is, cardamonin, pinostrobin chalcone, wogonin, chrysin, and pinocembrin, could suppress VEGF-induced angiogenesis as detected in the mouse aortic ring assay, and cardamonin showed the strongest inhibitory activity. Our further study demonstrated cardamonin remarkably suppressed VEGF-induced proliferation and migration, by decreasing the phosphorylation of ERK and AKT induced by VEGF with a dose-dependent manner in HUVECs [14]. Although these studies presented interpretation for cardamonin-mediated antiangiogenesis, deeper insights for the mechanisms involved are still appreciated.

MicroRNAs (miRNAs) are a new class of small non-protein-coding RNAs which are approximately 19–22 bases in length and that control gene expression posttranscriptionally by binding to various mRNA targets, predominantly in the 3′ untranslated region [18]. They control a wide range of biological functions such as cell proliferation, differentiation, apoptosis, and angiogenesis in endothelia cells [19–21]. These reports indicate that miRNAs might be involved in cardamonin-induced regulation of angiogenesis. In the present study, we investigated whether or not miRNAs have potential effects on cardamonin-induced inhibition of proliferation, migration, and tube formation in HUVECs induced by VEGF.

## 2. Materials and Methods

### 2.1. Cell Culture and Chemicals

HUVECs were purchased from Shanghai Institute of Cell Biology at the Chinese Academy of Sciences (Shanghai, China), cultured in RPMI1640 (Gino Biomedical Technology, Hangzhou, Zhejiang, China), and supplemented with 10% heat inactivated fetal bovine serum (Tianhang Biological Technology, Huzhou, Zhejiang, China). All cells were incubated at 37°C in humidified 5% CO_2_ and 95% air atmosphere. Cells at passages 3 to 8 were used in all experiments. Cardamonin was purchased from the National Institute for the Control of Pharmaceutical and Biological Products (Beijing, China).

### 2.2. Choosing miRs for Screening

Recent literatures revealed that a total of 17 miRs and 1 miR cluster had been reported to have connections with endothelial functions [22, 23]. We selected 14 of these miRs which might have been involved in regulation of angiogenesis, including miR-17-5p, miR-19a, miR-23a, miR-24, miR-31, miR-34a, miR-126, miR-130a, miR-132, miR-16, miR-21, miR-217, miR-221, and miR-378 for our study. Among them, miR-217, miR-221, and miR-378 were excluded out from our further work due to the nonspecificity of the primers that were used in the quantitative RT-PCR (qRT-PCR).

### 2.3. qRT-PCR

After being incubated with 8 ng/mL VEGF, HUVECs were treated with 50 *µ*M cardamonin for 3 h, 6 h, and 24 h, respectively. The total RNA was extracted with Trizol (Invitrogen, Carlsbad, California, USA), using the standard method. cDNA synthesis was performed with 1 *µ*g of total RNA, using the One Step PrimeScript miRNA cDNA Synthesis Kit (TaKaRa, Dalian, China) according to the manufacturer's instructions. qRT-PCR was performed on the ABI 7500 cycler (Applied Biosystems, Foster city, California, USA), using the miScript SYBR Green PCR Kit (TaKaRa, Dalian, China) according to the manufacturer's protocol. RNU6B (U6) was used as the endogenous control. The primers were designed using the untranslated sequences at 5′ and 3′ region which can be found in Table 1. The antisense primers were provided by miScript SYBR Green PCR Kit (TaKaRa, Dalian, China).

### 2.4. Small RNA Transfection

HUVECs were seeded for 24 h prior transfection when they reached 60–80% confluence. miR-21 inhibitors or miR-21 mimics, obtained from Ambion (Austin, Texas, USA), were transfected into HUVECs with the Lipofectamine RNAiMAX Reagent (Invitrogen, Carlsbad, California, USA) at a final concentration of 30 nM. A nonsilencing siRNA was used as a negative control. Cells were incubated with small RNA complexes for 24 h before the medium was changed.

### 2.5. Cell Proliferation Assay

HUVECs in 96-well plate were transfected with miR-21 mimic, miR-21 mimic negative control, miR-21 inhibitor, and miR-21 inhibitor NC and were allowed to grow for 24 h. After medium was changed, VEGF (8 ng/mL) [14] was added into the wells with or without 50 *µ*M cardamonin except for the control group, and the incubation was continued for another 24 h. Cell proliferation was assayed by 3-(4,5-dimethylthiazol-2-yl)-5-(3-carboxymethoxyphenyl)-2-(4-sulfophenyl)-2H-tetrazolium (MTS, Promega, Beijing, China) test according to the manufacturer's protocol. The absorbance at 460 nm was determined with a microplate reader (Bio-Rad, San Francisco, California, USA). For each group, 5 duplicate wells were detected per experiment. Cell nuclei were stained with Hoechst 33342 and observed with an inverted fluorescent microscope (Olympus, Tokyo, Japan).

### 2.6. Wound Healing Assay

An* in vitro* wound healing assay was performed to measure the unidirectional migration in HUVECs. HUVECs (2 × 10^4^ cells/mL) were seeded into 24-well plates. Cells were allowed to grow for 24 h after being transfected with miR-21 mimic, miR-21 mimic negative control (NC), miR-21 inhibitor, and miR-21 inhibitor NC RNAs and the monolayers of HUVECs were scratch-wounded to a 1 mm depth in a straight line using a 10 *μ*L pipette-tip when cells were at 70–80% confluence. VEGF (8 ng/mL) was added into the medium with or without 50 *µ*M cardamonin, and the incubation was continued for another 24 h. Images were photographed both immediately after wounding and after 24 h incubation, using a phase-contrast microscope (Olympus, Tokyo, Japan). Migration was calculated as the area of HUVECs that migrated from the injured edge into the wound zone. At least four points in each of three random fields were examined for each of three independent wounds.

### 2.7. Tube Formation

Tube formation by endothelial cells is a key step in angiogenesis which has several types of cells such as endothelia cells and pericytes participate [24]. Therefore, we investigated how cardamonin regulates capillary tube formation by HUVECs. HUVECs were cultured and transfected as described above. 24 h after transfection, cells were harvested and diluted to 2 × 10^5^ cells/mL in 0.5% FBS containing 20 ng/mL VEGF [25] and with or without 50 *µ*M cardamonin and seeded into matrigel-coated 24-well plate. After being cultured at 37°C for 8 h, the branch points of the capillary-like tubes were counted under a light microscopy (Olympus, Tokyo, Japan) at 100 magnification for three random fields [25].

### 2.8. Statistical Analysis

Data were given as mean ± SD. Statistical analysis was performed using one-way ANOVA and Dunnett's post-hoc test, with *p* < 0.05 being considered to indicate statistical significance.

## 3. Results

### 3.1. miR-21 Expression Was Downregulated in HUVECs Induced by VEGF after Cardamonin Treatment

The expression of 11 miRs associated with angiogenesis was quantitatively analyzed after treatment with VEGF (8 ng/mL) and cardamonin (50 *µ*M) for 3 h, 6 h, or 24 h, respectively. We found that cardamonin suppressed the expression for most of the miRs at 6 h and 24 h (Table 2). As noted, miR-21 was suppressed the most among these 11 miRs (with 58% inhibition compared with VEGF alone) (Table 2). The suppression was further down by 71% at 24 h compared with VEGF alone as determined by qRT-PCR. Other miRNAs, such as miR-23a, miR-31, miR-132, or miR-16, were also significantly downregulated with cardamonin treatment for 24 h compared to that of treatment for 3 h.

### 3.2. Transfection with miR-21 Mimics or Inhibitors Aggravates or Attenuates the Intracellular Level of miR-21

**Figure 1:**
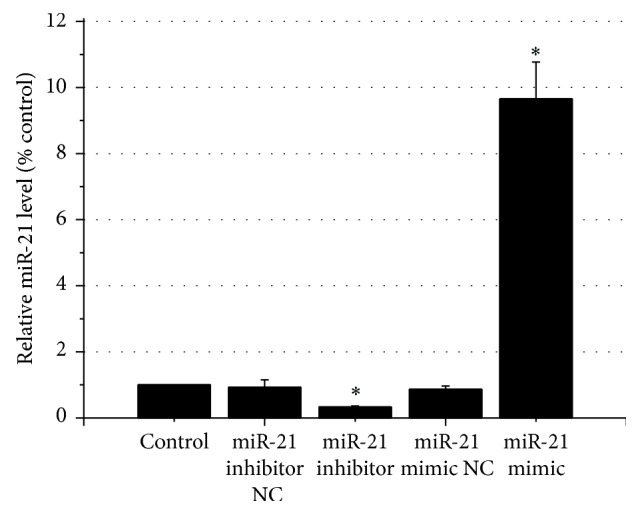
Relative miR-21 levels after transfection. Note: relative miR-21 lever was determined 24 h after transfection. ^*∗*^ *p* < 0.01 as compared with the control.

Since most of miRs have been downregulated and miR-21 was strongly suppressed by cardamonin, we used miR-21 mimics and miR-21 inhibitors to test the function of cardamonin on HUVECs. As shown in Figure 1, expression of miR-21 of HUVECs was augmented when cells were transfected with 30 nM miR-21 mimics, while expression of miR-21 was significantly reduced when transfected with 30 nM miR-21 inhibitors. None of the negative control RNAs had a significant effect.

### 3.3. Cardamonin-Mediated Inhibition of HUVECs Proliferation through the Expression of miR-21

**Figure 2:**
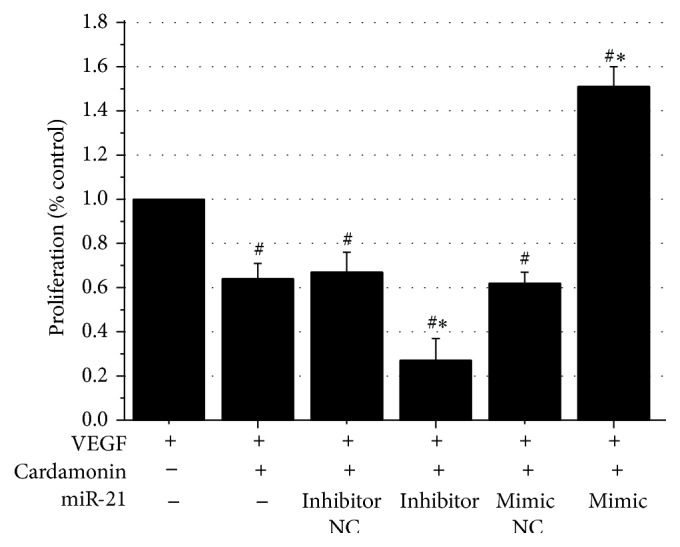
Cardamonin-mediated inhibition of HUVECs proliferation (means ± SD). Note: HUVECs were treated with VEGF (control), VEGF + cardamonin (50 *µ*M), or VEGF + cardamonin (50 *µ*M) + miRs. ^#^ *p* < 0.01 as compared with the VEGF group, ^*∗*^ *p* < 0.01 as compared with the VEGF + cardamonin group.

The cell viability was detected by the MTS test, which can reflect the proliferative ability of the HUVECs stimulated by VEGF. When cardamonin was added into the medium, HUVECs viability was significantly reduced (Figure 2). To test whether the inhibitory effect was associated with miR-21, miR-21 inhibitor was added together with cardamonin. Lower expression level of miR-21 caused a significantly stronger inhibition of HUVECs proliferation by 57.8% compared with cardamonin alone. To test whether cardamonin' inhibitory effect could be stopped by increasing the expression of miR-21, miR-21 mimic was given together with cardamonin. As show in Figure 2, cardamonin's inhibition was completely inversed. HUVECs proliferation was 151.0% of VEGF. These data suggested that cardamonin inhibited HUVECs by downregulation of miR-21. The excessive expression of miR-21 induced by transfection of miR-21 completely eliminated cardamonin's action and showed a higher proliferation above its basal level (stimulated by VEGF). The negative control RNAs for both the mimic and the inhibitor had no significant effects on cell proliferation (Figure 2).

### 3.4. Inhibitory Effect of Cardamonin on Endothelial Migration in HUVECs Associated with Downregulation of the Expression Level of miR-21

**Figure 3:**
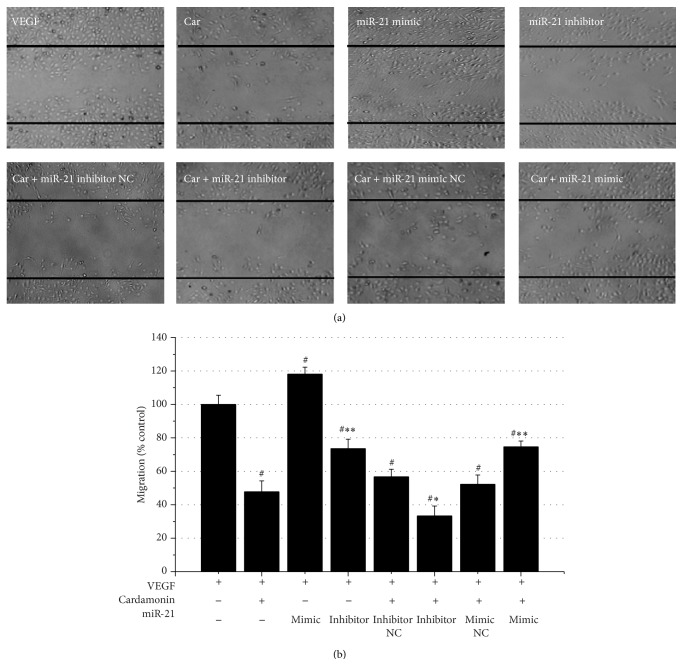
Cardamonin-mediated inhibition of HUVECs migration. Note: HUVECs in a six-well plate were photographed at the 0 h and 24 h time points. The areas of cells that migrated to the wound zones were measured separately with Image-Pro Plus 6.0 software. The experiment was repeated 3 times. (a) Cells in a six-well plate were photographed at 24 h time points. (b) The areas of cells that migrated into the wound zones, after treated with VEGF (control), VEGF + miR-21s, VEGF + cardamonin, or VEGF + cardamonin + miR-21s. ^#^ *p* < 0.01 as compared with the VEGF group, ^*∗*, *∗∗*^ *p* < 0.05 and 0.01, respectively, as compared with the VEGF+ cardamonin group.

In previous study, we found that cardamonin significantly inhibited HUVECs migration [14]. The migration of HUVECs was examined with a wound healing assay in this study. As shown in Figure 3, cardamonin significantly inhibited HUVECs migration by 65%. Because cardamonin caused downregulation of miR-21, we hypothesized that this inhibition was associated with the expression of miR-21. When miR-21 expression was decreased by transfection of miR-21 inhibitors, the migration inhibition was further downregulated by another 12.6%. When miR-21 expression was upregulated by mimic treatment, the mobility of HUVECs increased significantly by 15.4%. Neither the miR-21 mimic NC nor miR-21 inhibitor NC had a significant effect on the regulation of cardamonin-inhibited HUVECs mobility (Figure 3). To understand the effect of miR-21 on HUVECs migration, miR-21 mimic and miR-21 inhibitor were transfected; the results indicated that miR-21 overexpression can significantly increase HUVECs migration (118.2%) induced by VEGF; otherwise, HUVECs migration was remarkably inhibited by 26.4%, which confirmed the importance of miR-21 on HUVECs migration.

### 3.5. Inhibitory Effect of Cardamonin on Tube Formation in HUVECs Associated with Downregulation of the Expression of miR-21

**Figure 4:**
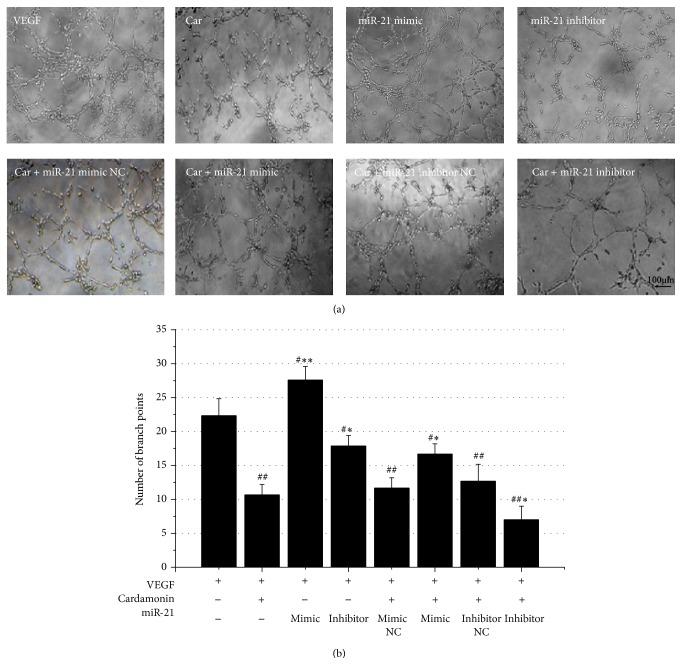
Cardamonin-mediated inhibition of HUVECs tube formation. Note: (a) HUVECs in a six-well plate were photographed at the 24 h time points. (b) The number of branch points after treated with VEGF, VEGF + cardamonin, VEGF + miR-21s, or VEGF + cardamonin + miR-21s. ^#, ##^ *p* < 0.05 and 0.01, respectively, as compared with the VEGF group, ^*∗*, *∗∗*^ *p* < 0.05 and 0.01, respectively, as compared with the VEGF + cardamonin group.

New capillary formation is required for the initial steps of angiogenesis, which involves processes such as endothelial cell proliferation and migration [26]. To investigate whether the inhibitory effects of cardamonin is associated with miR-21 expression, we evaluated the effects of cardamonin on VEGF-induced tube formation in HUVECs after being transfected with miR-21 mimic. Transfection with miR-21 mimic significantly stimulated the formation of capillary-like structures, when compared to VEGF treatment alone, and this action was significantly suppressed by transfection with miR-21 inhibitor alone; these data suggested that miR-21 plays an important role during angiogenesis. However, cardamonin treatment alone could also significantly suppress the formation of capillary-like structures by HUVECs, as compared to VEGF treatment alone, and cardamonin cotreatment with miR-21 inhibitor could further inhibit tube formation, and this could be remarkably reversed by cotreatment cardamonin with miR-21 mimic. Neither the miR-21 mimic NC nor the inhibitor NC had a significant effect on the regulation of cardamonin-inhibited tube formation (Figure 4). These data reconfirmed that cardamonin inhibits tube formation in HUVECs associated with downregulation of the expression of miR-21.

## 4. Discussion

Angiogenesis is a complex process of generating new capillary blood vessels. Regulation of angiogenesis includes endothelial cell proliferation, migration, basement membrane breakdown, and couple growth factors involved such as VEGF and FGF-2. Inhibition of endothelial cell proliferation and migration might contribute to the antiangiogenic activity. In our previous study, we found cardamonin remarkably suppressed VEGF-induced angiogenesis as detected in the mouse aortic ring assay. Cardamonin also obviously suppressed proliferation and migration induced by VEGF with a dose-dependent manner in HUVECs [14]. miRs are short noncoding RNAs function as negative regulators of gene expression which have been regulators of angiogenesis. Herein, we found for the first time that miR-21 was downregulated strikingly after treatment with cardamonin (50 *µ*M) in a time dependent manner.

As miR-21 is frequently overexpressed in human cancers and acts as oncogene and is involved in promoting cell proliferation, invasion, and migration [27–29], it may also play pivotal role in endothelial cell proliferation and migration and influence angiogenesis. Guduric-Fuchs et al. reported that downregulation of miR-21 reduced the proliferation, migration, and tube-forming capacity of retinal microvascular endothelial cells (RMECs) [30]. Zhao et al. evidenced that miR-21 can mediate arsenite induced HUVEC upregulation VEGF and promote angiogenesis [31]. In our present study, we found VEGF could upregulate miR-21 expression, and overexpression miR-21 could significantly enhance VEGF-induced HUVEC migration and tube formation, which confirmed the importance of miR-21 on angiogenesis.

Concerning the mechanism of cardamonin on antiangiogenesis, miR-21 mimics and inhibitors were transfected on HUVEC. Results indicated that treatment with miR-21 mimics abrogated the cardamonin-mediated inhibition of HUVEC proliferation, migration, and angiogenesis, while treatment with miR-21 inhibitors aggravated the cardamonin-mediated inhibition of HUVEC proliferation, migration, and angiogenesis, indicating that miR-21 might play a vital role in cardamonin-induced inhibition of angiogenesis triggered by VEGF.

It is noted that other miRNAs, for example, miR-132 and miR-16, were also obviously downregulated after treatment with 50 *µ*M cardamonin. It is reported that miR-132 plays an important role in angiogenesis in infectious ocular disease [32] and miR-16 affects the angiogenesis by targeting VEGF [33, 34]. Therefore, their roles in regulation of cardamonin-induced antiangiogenesis should be addressed in future studies.

Our previous study showed that cardamonin decreased the phosphorylation of ERK and AKT induced by VEGF with dose-dependent manner in HUVECs [14]. It is suggested that miR-21 induces angiogenesis and promotes carcinoma progression through AKT and ERK pathways [35, 36]. Thus, the interactions between miR-21 and AKT and ERK pathways require further study.

## Figures and Tables

**Table 1:** The list of primers used in qRT- PCR.

	Sense primers	Antisense primers
miR-17-5p	CAAAGTGCTTACAGTGCAGGTAG	Provided by miScript SYBR Green PCR Kit (TaKaRa, Dalian, China)
miR-19a	TGTGCAAATCTATGCAAAACTGA	
miR-23a	CACATTGCCAGGGATTTCC	
miR-24	GCTCAGTTCAGCAGGAACAGA	
miR-31	GCAAGATGCTGGCATAGCT	
miR-34a	GGCAGTGTCTTAGCTGGTTGT	
miR-126	TCGTACCGTGAGTAATAATGCG	
miR-130a	CAGTGCAATGTTAAAAGGGCAT	
miR-132	TAACAGTCTACAGCCATGGTCG	
miR-16	TAGCAGCACGTAAATATTGGG	
miR-21	ACGTTGTGTAGCTTATCAGTG	
RNU6B	CTCGCTTCGGCAGCACA	AACGCTTCACGAATTTGCGT

**Table 2:** Expression levels of miRNAs after cardamonin treatment (means ± SD).

Treatment		miR	Fold change		
VEGF	Car		3 h	6 h	24 h
+	−		1.00	1.00	1.00
+	+	miR-17-5p	1.31 ± 0.45	0.96 ± 0.32	0.45 ± 0.10^*∗∗*^
+	+	miR-19a	0.85 ± 0.15	0.70 ± 0.23^*∗*^	0.32 ± 0.06^*∗∗*^
+	+	miR-23a	1.88 ± 0.69^*∗*^	0.50 ± 0.12^*∗∗*^	0.35 ± 0.13^*∗∗*^
+	+	miR-24	0.75 ± 0.18	0.46 ± 0.17^*∗∗*^	0.42 ± 0.06^*∗∗*^
+	+	miR-31	1.33 ± 0.53	0.78 ± 0.12^*∗*^	0.36 ± 0.11^*∗∗*^
+	+	miR-34a	1.19 ± 0.62	0.50 ± 0.09^*∗∗*^	0.43 ± 0.07^*∗∗*^
+	+	miR-126	1.23 ± 0.65	0.55 ± 0.06^*∗∗*^	0.65 ± 0.20^*∗*^
+	+	miR-130a	0.93 ± 0.48	0.52 ± 0.17^*∗∗*^	0.72 ± 0.26^*∗*^
+	+	miR-132	1.10 ± 0.59	0.61 ± 0.17^*∗∗*^	0.39 ± 0.06^*∗∗*^
+	+	miR-16	1.29 ± 0.61	0.63 ± 0.19^*∗∗*^	0.37 ± 0.09^*∗∗*^
+	+	miR-21	1.11 ± 0.62	0.42 ± 0.16^*∗∗*^	0.29 ± 0.12^*∗∗*^

Note: ^*∗*,*∗∗*^ *p* < 0.05 and 0.01, respectively, compared with the VEGF group. miRNA level was determined by qRT-PCR after HUVECs were treated with VEGF and cardamonin for 3, 6, and 24 hours. Data was expressed as fold change of VEGF group.
